# Postoperative Care of the Transplanted Patient

**DOI:** 10.2174/157340311797484286

**Published:** 2011-05

**Authors:** Kurt R Schumacher, Robert J Gajarski

**Affiliations:** University of Michigan Congenital Heart Center

**Keywords:** Pediatric heart transplant, critical care.

## Abstract

The successful delivery of optimal peri-operative care to pediatric heart transplant recipients is a vital determinant of their overall outcomes. The practitioner caring for these patients must be familiar with and treat multiple simultaneous issues in a patient who may have been critically ill preoperatively. In addition to the complexities involved in treating any child following cardiac surgery, caretakers of newly transplanted patients encounter multiple transplant-specific issues. This chapter details peri-operative management strategies, frequently encountered early morbidities, initiation of immunosuppression including induction, and short-term outcomes.

## INTENSIVE CARE MANAGEMENT

### Hemodynamic Instability

Adequate monitoring of the post-operative heart transplant patient is essential. Recently published International Society for Heart and Lung Transplantation (ISHLT) guidelines for the peri-operative monitoring of both adult and pediatric heart transplant recipients are detailed in Table **1** [1]. Of these, standard pediatric monitoring would include all except assessment of pulmonary arterial wedge pressure (PAWP) and cardiac output via Swan-Ganz catheters due to concerns of catheter size and maintaining appropriate catheter position especially in smaller recipients. However, continuous, direct measurement of pulmonary artery pressures is often monitored in pediatric patients, particularly those with elevated pre-transplant pulmonary arterial pressures.

Peri-operative hemodynamic instability can be present and is secondary to multiple causes including graft reperfusion injury, post-bypass inflammatory response, elevated pulmonary vascular resistance, and labile fluid status. Vasoactive pharmacologic support is routinely necessary to augment marginal cardiac output mediated by ventricular dysfunction and associated systemic hypotension. The catecholamine stores of the newly transplanted heart are often depleted necessitating exogenous catechol supplementation [2]. Continuous infusions of isoproterenol, dobutamine, dopamine, and low-dose epinephrine all increase left ventricular contractility. These agents can be similarly used to improve right ventricular function. Alpha-adrenergic agonists including high-dose epinephrine (>0.05mcg/kg/min), phenylephrine, and norepinephrine may be used to achieve adequate systemic perfusion pressure when necessary. With limited supporting adult data, because of its specific peripheral vasoconstricting properties, vasopressin has been recommended as treatment for vasodilatory shock and may be considered when α-agonists have been ineffective in combating refractory low systemic vascular resistance [1]. If significant hemodynamic instability is present and unresponsive to standard pharmacologic interventions, direct surgical exploration to assess for and treat potential cardiac tamponade should be strongly considered. Hyperacute (pre-formed antibody mediated) rejection may also be an etiology for cases of profound hemodynamic instability [1] and often requires aggressive therapies including mechanical circulatory support and plasmapheresis for reversal.

As mentioned above, when pharmacologic treatment alone is inadequate to support the failing graft, mechanical circulatory support may be required. The ISHLT recommendations state that extra-corporeal membrane oxygenation (ECMO) is the first choice for support in the setting of primary graft failure, and ECMO should be instituted immediately in pediatric patients with progressive post-operative allograft dysfunction [1]. Several previous reports have shown that this modality has been used successfully to salvage severe graft failure following pediatric transplantation [3] [4]. Tissot and colleagues reported that 9% of their transplant recipients required peri-operative ECMO for right ventricular or biventricular failure, and/or hyperacute rejection. Of those, 54% survived to hospital discharge, and, importantly, the long-term survival of this group was not significantly different from a non-ECMO comparison cohort. These investigators also found that, while pre-transplant cardiac diagnosis was not a risk factor, longer total ischemic time, younger age and lower weight at transplant were associated with an increased risk for peri-operative ECMO support [3]. 

### Bleeding and Volume Status

Post-operative bleeding can be significant in children following heart transplantation. Causes are multi-factorial and include previous congenital cardiac surgery necessitating extensive dissection, cardiopulmonary bypass, multiple suture lines, pre-transplant heparinization for VAD or ECMO support, and poor pre-operative nutritional status. Platelet and fresh frozen plasma infusion should be used as necessary to control hemorrhage. Recombinant factor VII may be useful for refractory bleeding [1]. Volume resuscitation including packed (preferably leukocyte reduced if not CMV negative) red blood cells may be necessary, but should be administered with caution given the increased risk for allosensitization from transfused leukocytes which may express non-donor matched HLA antigens. Patients with refractory hemorrhage or those demonstrating clinical evidence of cardiac tamponade should be surgically explored. 

Fluid management may be challenging. Care must be exercised to provide adequate cardiac filling without causing substantial overload to the already stressed right ventricle. Goal central venous pressures of 5-12mmHg should be targeted [1]. Fluid resuscitation with colloid should be performed when necessary to maintain adequate filling. Fluid overload can also occur following volume resuscitation for hypotension and associated capillary leak after surgery and is typically managed with intermittent or continuously infused loop diuretics and adjunctive use of thiazides when needed. Acute renal failure occurs post-operatively in 3-10% of transplant recipients [5]. Hemodialysis may be necessary for refractory fluid overload and oliguria in the presence of a rising serum creatinine. Multi-disciplinary team management including nephrology is often useful in this circumstance. 

### Hypertension

Patients often develop systemic hypertension in the immediate post-operative period. This can be secondary to baroreflex-mediated hypertension, catecholamine dysregulation from low cardiac output pre-transplant, significant pre-existing renal injury, and newly initiated immunosuppressive medications such as corticosteroids or calcineurin inhibitors [6]. Heart-transplant recipients can also develop salt-sensitivity related to blunted diuretic and natriuretic responses caused by a failure to suppress fluid regulatory hormones. This may be due to altered cardiorenal-neuroendocrine interactions in the context of a denervated graft [7]. Systemic hypertension should be treated to minimize afterload on the graft left ventricle (LV). Calcium-channel blockers, angiotensin converting enzyme inhibitors or a combination of both usually provide adequate blood pressure control.

### Donor Size Mismatching

Recipient/donor size mismatching can also influence post-operative course. “Big heart syndrome” results when the donor size is significantly larger than the recipient. In the early transplant period, donor/recipient weight ratio mismatches of >2.0 may result in systemic hypertension syndrome with associated central nervous system symptoms including seizures and coma. Treatment consists of anti-hypertensive medication titration to achieve a normal blood pressure for age [8]. An inappropriately small donor heart size has been associated with increased mortality, and a donor/recipient weight ratio <1 has been reported as a significant predictor of fatal post-operative heart failure [9].

Post-operative pericardial effusions develop in 9-21% of adult recipients [10, 11]. The incidence in pediatric patients is unknown but likely similar to adults and may, in part, be related to an increased pericardial volume created after a dilated heart is replaced with normal-sized heart that fills with fluid. Unless the effusion is hemodynamically compromising or there is a strong suspicion of an infectious etiology, the effusion does not require surgical or percutaneous drainage and can be monitored serially by echocardiography [1]. Hemodynamic instability in the presence of an effusion warrants surgical or catheter-based drainage. 

### Arrhythmias

Post-transplant sinus node dysfunction is common with a reported prevalence as high as 44% [12] and is likely related to myocardial ischemia and surgical manipulation [13] [14]. Due to diastolic dysfunction and impaired filling of the transplanted heart, atrio-ventricular (AV) synchrony and adequate heart rate are often required to maintain cardiac output. A continuous isoproterenol infusion is often used as a chronotrope to increase heart rate. Alternatively, either AV pacing or, with intact AV node conduction, atrial pacing alone may be performed via temporary pacing wires placed at the time of surgery. The ISHLT guidelines recommend pharmacologic treatment or pacing to maintain a minimum heart rate of 90 beats/min [1]. This rate can be increased for effect, and higher rates should be targeted for smaller patients. Although sinus node dysfunction is typically transient [12], some patients will have permanent sinus node dysfunction and require permanent pacing. A 2-5% prevalence of pacemaker placement has been reported with sick sinus syndrome and complete heart block constituting the most common indications [14] [15]. 

## PULMONARY VASCULAR RESISTANCE

As discussed in another chapter*, *elevated pulmonary vascular resistance (PVR) is a significant risk factor for early post-transplant right heart failure and mortality in both pediatric and adult patients and is often multi-factorial in origin [16] [17] [18] [19]. Several studies have demonstrated an RV failure risk up to 75% with a 15% mortality risk among patients with pre-transplant PVRi (indexed to body surface area) >6 Wood units x m^2^. This contrasts with a 20% risk of RV failure in patients without increased PVR [20] [21] [17]. The mechanism of RV failure in the immediate post-OHT period is multifactorial. The donor heart, already exposed to donor-related myocardial strain, ischemia, cardioplegia, and surgical trauma, is then exposed to elevated recipient PVR often complicated by bypass-induced transitional pulmonary vascular hyper-reactivity [22]. This sudden and dramatic increase in PVR can cause rapid, and possibly irreversible, RV failure. 

Given this risk, particular attention must be paid to adequate control of the post-operative PA pressures. An indwelling pulmonary arterial line will permit continuous post-operative pulmonary arterial pressure monitoring and facilitate treatment when elevated in an effort to avoid strain-related RV myocardial failure. Conventional treatment options in this setting have included increased sedation with neuromuscular blockade if necessary, avoidance of hypercapnia and hypoxia, and serum alkalinzation (pH 7.45-7.55). 

Over the last decade, treatment with pulmonary vasoactive substances has been used as first-line therapy or in conjunction with the above-listed maneuvers. Nitric oxide (iNO) is an inhaled pulmonary vasodilator that rapidly and effectively reduces PVR and RV pressure in the post-transplant patient [23] [24]. Nitric oxide may be started intra-operatively when significant RV dysfunction or failure is first recognized, and some centers empirically treat all patients with pulmonary vasodilators prior to weaning from cardiopulmonary bypass as recent studies have shown this strategy decreases the incidence of post-operative right heart failure [25]. Often, iNO is continued for several days post-operatively when RV failure is a concern [17]. Despite its efficacy, iNO is not a long term treatment option due to its toxicities (i.e. methemoglobinemia), need for continuous delivery, and considerable cost which have motivated investigators for explore alternative agents to treat post-operative pulmonary hypertension. 

Iloprost is an aerosolized prostacyclin derivative and potent pulmonary vasodilator. When inhaled, it preferentially dilates local vascular beds in well ventilated areas of the lung and has been effective in treating primary pulmonary hypertension in adults [26]. While experiential data is limited, iloprost has been effective in decreasing pulmonary vascular resistance in a small series of adult post-transplant recipients [27, 28], and comfort with its use in pediatrics is growing. 

Sildenafil, a phosphodiesterase type-5 inhibitor in pulmonary vascular smooth muscle, has therapeutic effects within hours of dosing and has been effective in reducing pulmonary artery pressures [20, 29]. Daftari and colleagues (2010) reported their success with initiating sildenafil in combination with other pulmonary vasodilators in the immediate post-transplant period for 7 patients with pre-operative PVR >6 Wood units. Prior to transplant, all patients had been treated with sildenafil, bosentan, or both. Post-operative sildenafil therapy, and in some cases additional pulmonary vasodilator therapy, was continued until PVR normalized. All 7 patients were reportedly alive at 1 year post-transplant. 

## PRIMARY GRAFT FAILURE

Primary graft failure is defined as early post-transplant cardiac failure without an identifiable immunologic or anatomic etiology [19, 22, 30] and has been reported in 20%-30% of patients within the first 30 days post-transplant [19] [31]. The ISHLT reported >20% of deaths within 30 days of transplant were due to primary graft failure, making it the leading cause of peri-operative mortality [32]. In infants, more than 50% of deaths in the first post-transplant month have been attributed to primary graft failure [33]. Recipient congenital heart disease, pre-transplant mechanical support, increasing donor ischemic time, anoxia-mediated donor death, prolonged donor resuscitation time, and increasing donor:recipient weight ratio have all been associated with increased graft failure risk [19], while donor blood type O+ and donor hyperdynamic systolic function are protective [19]. When present, treatment is symptomatic and directed at increasing contractility as well as minimizing afterload and pulmonary vascular resistance. ECMO should be promptly instituted when standard post-operative pharmacologic treatment is inadequate. Given the risk of graft loss and mortality coupled with the known risk factors for primary graft failure, the importance of rigorous patient and donor selection criteria cannot be overstated.

## ABO INCOMPATIBLE TRANSPLANTATION

ABO incompatible (ABOi) transplantation has been detailed in a separate section. However, some specific issues are particularly relevant in the immediate post-operative care of ABOi recipients and are, therefore, worthy of reiteration. Special transfusion protocols must be used peri-transplant to avoid early and late donor blood group sensitization Table **2** [34]. Although the risk of isohemagluttinin antibody mediated rejection is low by published reports [35], isohema-gluttinin mediated rejection must be considered a potential cause of any acute graft failure. In Dipchand and colleagues’ review of their extensive experience with ABOi transplantation, donor specific isohemagluttinin antibodies developed in 2/35 patients, neither experienced significant graft dysfunction, and, with therapy, these antibodies disappeared within 8 weeks and did not recur [35]. Roche and colleagues (2008) similarly found low rates of cellular rejection in their ABOi patient cohort (n=21) [36]. For surveillance and assurance of adequate treatment in patients who do develop donor specific isohemagluttinin antibodies, serum titers must be collected routinely in the immediate post-transplant period.

Standard immunosuppression regimens are used in ABOi transplants with no increased risk of rejection compared to ABOc patients [35, 36]. 

## INFECTION

Infections occur in up to 25% of pediatric recipients during the early post-operative period, and 60% of these infections are bacterial [37]. The most common bacterial pathogens reported are Staphylococcus, Pseudomonas, and Enterobacter cloacae [38]. Blood stream and pulmonary infections are most common followed by urinary tract and surgical site infections [39, 40]. The ISHLT guidelines recommend peri-operative antibiotic prophylaxis against skin flora, particularly Staphylococcus aureus, and, if donor infection is confirmed, additional targeted therapy against the potentially transmittable donor organism should be strongly considered [1].

Up to 7% of children may develop a peri-operative fungal infection following transplant [39]. This infection type is often localized, but disseminated disease can occur and is associated with an increased risk of death during the first year post-transplant [41]. The most common pathogens are Candida species and molds, specifically Aspergillus [37]. Initiation of antifungal prophylaxis with nystatin or clotrimazole is recommended after extubation [1]. 

Pneumocystis jiroveci infection has been reported in approximately 4% of adult heart transplant recipients and presents with acute and potentially fatal systemic illness [42]. There have been fewer confirmed infections in pediatric patients, but because of its virulence in immunosuppressed patients, current recommendations are for 3-24 months of post-transplant prophylaxis with trimethoprim/sulfamethoxazole, *or* aerosolized pentamidine isethionate, pyrimethamine or dapsone (with or without trimethoprim) in patients with sulfa allergies [1].

Cytomegalovirus (CMV) infection is the most common infectious agent identified after pediatric transplant, and the incidence in post-transplant patients who have not received CMV prophylaxis is 60-90% [37, 43]. In contrast to adults in which previous CMV exposure is common, fewer pediatric donors and recipients are CMV seropositive (CMV+) increasing the risk of CMV infection in pediatric recipients [44]. Often this infection consists of benign viremia and does not lead to clinically relevant disease [44]. However, up to 18% of pediatric CMV-mismatched patients (R-/D+) develop clinical CMV disease with typical findings of fever, appearance of atypical lymphocytes, lymphopenia, myalgias, arthralgias, thrombocytopenia, and renal impairment; severe manifestations of disease may include interstitial pneumonia, esophagitis, gastritis, colitis, retinitis, and encephalitis [44]. CMV+ recipients can also develop CMV disease, either from reactivation or new donor transmitted disease [43].

Because CMV disease can occur early after transplant and the peri-operative morbidity can be significant, prophylactic and pre-emptive strategies to minimize or prevent CMV infection/disease have been developed. Prophylaxis consists of intravenous (IV) ganciclovir or oral valganciclovir initiated in the early post-operative period with a goal of preventing CMV infection [45]. Pre-emptive therapy consists of close monitoring of recipient CMV status, either by quantitative DNA-PCR or CMV antigenemia, and initiating treatment when a previously CMV negative patient becomes CMV positive thereby minimizing transition of infection into significant CMV disease [45]. When both strategies were compared in a recent adult cohort study, prophylaxis was superior to pre-emptive therapy with a reduction in CMV infections, decrease in subsequent CMV disease, and reduction in coronary intimal thickening by intravascular ultrasound [46]. Prophylaxis with IV ganciclovir, oral valganciclovir, or CMV immunoglobulin (CytoGam) is commonly used by pediatric transplant centers for CMV-mismatched patients and has a survival benefit over non-prophylaxis [47]. Though not standard practice, post-operative dual-therapy with CytoGam and ganciclovir is effective both as preemptive and prophylactic therapy and has been shown to attenuate symptoms in active disease [43, 48, 49]. The recent ISHLT guidelines recommend initiating treatment with oral or IV ganciclovir or valganciclovir for CMV+ or CMV-mismatched pediatric recipients [1]. 

## REJECTION

Despite evolving immune therapies, rejection continues to be a major source of morbidity and mortality in the immediate post-operative period. Rejection is an adaptive immune response and, for discussion purposes, is usually divided into 2 forms: T-cell mediated and antibody (humoral) mediated. 

Acute cellular rejection is T-cell mediated and usually occurs after the first post-operative week. Many transplants recipients will experience some degree of ongoing non-damaging cellular rejection. This asymptomatic, mild rejection (ISHLT ≤1R) does not typically require treatment as there is frequent spontaneous resolution, and treatment of these episodes has not been associated with survival benefit [50, 51]. However, more significant treatable rejection also occurs, and nearly 40% of adult recipients have reportedly experienced as least one episode of grade ≥2R rejection in the first post-transplant year [32], with the highest incidences during the initial 3 months [52]. In recent years, however, incident treatable rejection has decreased, possibly due to novel immunosuppressive regimens or combinations; however, the incidence of rejection causing hemodynamic compromise and death has remained unchanged [53]. Rejection remains the primary cause in 10% of all mortalities within the first 30 days following transplant [32].

Biopsy-proven rejection grade ≥2R, with or without clinical symptoms, is medically treated by most transplant physicians. Pulsed intravenous corticosteroids are the usual initial treatment in the immediate post-operative period [51]. Lack of response to steroid treatment and/or progressive clinical deterioration can be treated with more aggressive cytolytic therapy, usually anti-thymocyte globulin [54].

Cellular rejection surveillance is determined by the patient’s overall risk for rejection and continues to be center dependent. Endomyocardial biopsy (EMB) is the gold standard for diagnosis [55]. Initial EMB is performed in older pediatric patients within the first 2 weeks after transplant [55, 56]. Infants, possibly due to the immaturity of their immune system, appear to be at decreased risk for rejection [57]. Many centers perform routine EMB on infants significantly less frequently or not at all, instead depending on physical exam and echocardiogram to aid in diagnosis, and biopsy only for clinical indications [58, 59]. With any clinical deterioration in the early post-operative period, evaluation of and treatment for rejection as the potential cause should be considered.

Humoral rejection results from an antibody-mediated response to mismatched human leukocyte antigens (HLAs) present within the donor myocardium and vascular endothelium, and the number of mismatches may influence the speed and degree of rejection [60]. Pathologically, this form is characterized by a lack of significant cellular rejection on EMB and is often accompanied by left ventricular dysfunction and detection of donor-specific antibody in the recipient serum [60] as discussed in another chapter. Initial treatment is similar to cellular-rejection and includes pulsed corticosteroids and cytolytic therapy (often anti-thymocyte globulin); however, adjunctive therapy to target B-cell activity and decrease circulating antibodies is also frequently used. Plasmapheresis and intravenous immune globulin G (IVIg) therapy have been employed to remove pre-formed antibody and are frequently used by pediatric transplant physicians [61-63]. Cyclophosphamide and mycophenolate mofetil have also been used to directly suppress B-cell populations and Rituximab, a monoclonal antibody directed against the B-cell CD20 receptor, has been successfully used to treat humoral rejection [64-66]. 

## ALLOSENSITIZATION

Genetically programmed human leukocyte antigens (HLA) are divided into 2 distinct classes (I and II) and expressed on all somatic cells giving each of us a distinct HLA signature. Immune recognition of “non-self” HLA antigens results in production of anti-HLA antibodies and is termed allosensitization. The most detailed assessment of anti-HLA antibody is currently performed via a panel reactive antibody (PRA) test using Luminex single-antigen bead technology. Allosensitization in children with congenital heart disease is particularly prevalent as these children are often exposed to many potential sensitizing events prior to transplant. In this group, production of anti-HLA antibody may occur during use of a ventricular assist device, prior blood transfusions (especially platelets), or following implantation of cryopreserved homografts which are used in many surgical reconstructions [67-69]. Interestingly, sensitization among infants and young children supported with extra-corporeal membrane oxygenation (ECMO) may occur less frequently than in children with ventricular assist devices [70]. Patients with PRAs >10% have an increased risk of rejection and lower graft survival rates [62, 71, 72]. Previously, these patients required prospective crossmatching, which necessitated transport of receipient serum to the donor center for reaction with donor serum prior to harvest, to identify a suitable organ. Because this process not only delayed donor allocation but also reduced the recipient’s potential donor pool due to distance restrictions, most centers now attempt pre-transplant desensitization. Although there is currently no universally accepted desensitization protocol, multiple agents have been used including oral methotrexate, cyclophosphamide and mycophenolate mofetil. Successful desensitization after use of these drugs was generally poor, and more recently, clinicians have implemented strategies using Rituximab and IVIg. IVIg is first administered to bind and remove circulating anti-HLA antibodies. Rituximab is then serially administered to suppress CD20+ B-cells and limit the future production of anti-HLA producing plasma cells, leading to a slow decrease in anti-HLA antibodies over time [73]. Small trials are ongoing to assess the effectiveness of this approach. Anecdotal reports of Bortezomib have suggested this agent may have additional efficacy over Rituximab, but randomized trials have not yet confirmed its utility. Bortezomib is a proteosome inhibitor that has been shown in pre-clinical studies to promote the emergence of regulatory T-cell populations that inhibit stimulated effector T-cells, thereby limiting the pathway ultimately producing anti-HLA antibodies [74]. There is very limited data on Bortezomib in heart transplantation, but small studies in renal transplant patients suggest it may have a role in desensitization [74]. Regardless of agent used, in most cases, desensitization will not be completely effective and will require virtual-crossmatching to maximize the success of transplanting a highly-sensitized patient. This technique enables the recipient center to remotely predict the risk of accepting a given donor since Luminex technology allows quantitative identification of potential donor-specific antibodies. This increases the center’s ability to select appropriately HLA-matched organs and minimize the risk of peri-operative antibody-mediated rejection. While longer-term data is not yet available, graft survival after virtual crossmatching is acceptable with low humoral rejection rates [70, 75]. 

## INDUCTION THERAPY

**Fig. (1):**
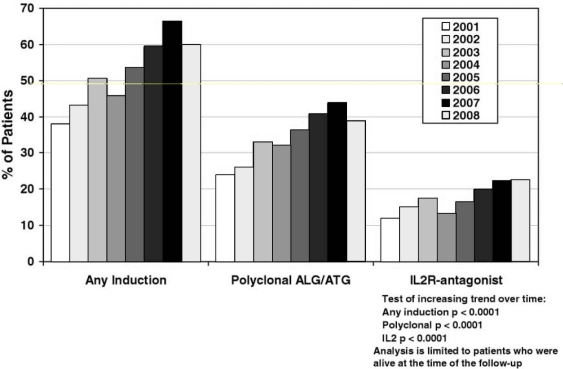
Induction immunosuppression for transplants performed January 2001–June 2009. ALG, anti-lymphocyte globulin; ATG, anti-thymocyte globulin; IL-2R, interleukin-2 receptor [32].

Induction therapy refers to the administration of a special group of immunosuppressant agents in the pre- and peri-operative period to rapidly disable the normal host response toward the transplanted graft [32]. Although there has been a recent trend toward increased usage, induction therapy is not considered a universal standard of care and only 50-70% of centers currently report its use (Fig. **1**) [76]. 

**Fig. (2):**
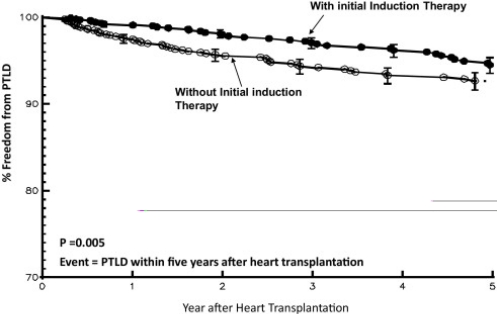
Curves represent freedom from PTLD stratified by receipt of induction. Note that induction therapy is associated with a higher freedom from lymphoma throughout the follow-up period.

**Fig. (3):**
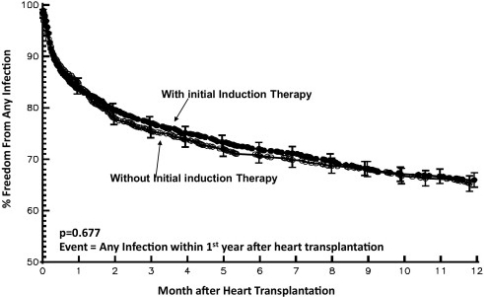
Kaplan-Meier curve of freedom from infection stratified by use of induction. No difference was identified in probability of infection based on induction (i.e. overall infection likelihood was not greater in patients receiving induction).

After their initial intent to induce “immune tolerance” failed, practical application of these drugs evolved into strategies to reduce early rejection, which was associated with improved peri-operative survival [77-81]. More recently, induction therapy has been used to delay the initiation of nephrotoxic calcineuin inhibitors and to achieve steroid avoidance [81-83]. Despite its success in achieving these goals, the universal use of induction therapy was nonetheless limited due to mixed reports that it may have increased the risk for infection and post-transplant lymphoproliferative disease (PTLD). Several adult studies found increased incidence of CMV and fungal infections [84, 85] or PTLD [86] while others found no association with these events [87, 88]. Likewise, pediatric patients are not universally induced due to similarly conflicting results [79, 89-91]. However, in a recent study of patients enrolled in the Pediatric Heart Transplant Study database, Gajarski and colleagues analyzed 2374 transplanted patients, 53% of whom received induction therapy. Despite use of variable induction agents (detailed below), the authors found that when compared to non-induced patients, the induction group had a significantly lower rate of PTLD and a similar infection rate. The authors concluded that induction strategies can be implemented without increasing early infection or PTLD risk (Fig. **2**, **3**) [92]. 

### Monoclonal Antithymocyte Antibody

Muromonab (OKT3) is a murine antibody targeting the human T-cell CD3 receptor which disrupts its ability to respond to an antigen challenge and leads to T-cell opsinization and removal by circulating macrophages [93]. Side effects include fever, rash, aseptic meningitis, and anaphylaxis. In addition, the opsinization of CD-3+ T-cells can lead to the release of multiple cytokines causing headache, nausea, vomiting, fever, chest pain, and dyspnea from pulmonary edema, collectively termed “cytokine release syndrome” [22, 93]. Compared with other agents, the recent mega-analysis found that OKT3 was the only induction agent associated with an increased risk for PTLD and infection with CMV or fungus [92]. Because of these side effects and unacceptable morbidities, OKT3 use has been largely abandoned in favor of alternative agents (listed below) with similar benefits but reduced adverse event profiles [94]. 

### Polyclonal Antithymocyte Globulin (ATG)

Two polyclonal antithymocyte antibody preparations are available (Table **3**). Equine ATG (ATGAM, Pharmacia Upjohn, Pfizer, NY) is purified after horse immunization with human T-cells, and rabbit ATG (Thymoglobulin, Genzyme Corporation, Cambridge, MA) is produced by rabbit immunization with human thymocytes. Both preparations lead to production of polyclonal antibodies against multiple human antigens including immune cell surface receptors and HLA antigens expressed on T-lymphocytes [94]. Injection of the polyclonal sera into humans leads to antibodies coating recipient immune cells, which results in rapid depletion of recipient T-lymphocytes via opsinization, phagocytic, and natural killer cell mechanisms [22, 94]. Individual transplant physicians dose these agents to target total lymphocyte counts of 0.1-0.3 (X10^3^/mm^3^) which appears efficacious without significantly increasing the risk of opportunistic infections [79, 81, 94]. Direct comparisons of the horse- and rabbit-derived preparations showed that the rabbit preparation more potently decreased circulating lymphocyte counts without a change in safety profile [95]. No increased risk of infection or PTLD has been documented after ATG administration in several recent reports [79, 89, 92].

### IL-2 Receptor Antagonists 

Use of novel interleukin-2 receptor antagonists (IL-2Ra) has increased over the last several years (Table **3**) [32]. These agents bind to the alpha-subunit of the IL-2 receptor on activated T-cells preventing T-cell proliferation and attenuating the graft-directed immune response [93]. There are two currently available forms utilized in pediatric transplantation: basiliximab (Simulect, Novartis, New Jersey, USA), a chimeric mouse/human antibody, and daclizumab (Zenapax, Roche Pharmaceuticals, New Jersey, USA), a humanized (>90% human, <10% mouse) antibody [94]. Several recent studies have shown IL-2R antagonists to be effective in reducing the incidence of acute rejection [53, 83, 96]. Although data evaluating these morbidities remains limited, compared to other induction agents, IL-2R antagonists may also be associated with a lower infection and PTLD event profile [97]. Interestingly, unlike other induction agents, basiliximab appears to confer better prevention of acute rejection when given prior to graft implantation [96]. Previously, clinicians believed that immunosuppressants given pre-operatively would be diluted or filtered from the circulation during bypass. However, because basiliximab rapidly binds to its target molecule, important loss of drug during bypass is unlikely and prevention of early T-cell activation may be the key to this drug’s efficacy [96]. Basiliximab and daclizumab have not been studied in head-to-head randomized cardiac studies, however, data from pediatric renal transplantation demonstrated equivalent efficacy and safety between the two agents [98]. 

## IMMUNE MAINTENANCE THERAPY

Unless delayed by use of induction, maintenance immunosuppression is routinely initiated within the first several post-operative days. Most maintenance regimens are triple-therapy based and include a calcineurin inhibitor, cell cycle inhibitor and corticosteroid.

### Calcineurin Inhibitors

Calcineurin inhibitors (CNI) are the crux of current maintenance therapy (Table **4**). CNIs inhibit the phosphatase action of calcineurin, a key enzyme in the production pathway of multiple cytokines including IL-2. This action inhibits the expansion of CD4+ and CD8+ cell lines and differentiation of CD4+ T-cell subsets [94]. Currently, 2 CNIs are frequently used, cyclosporine A (CSA, Neoral; Novartis Pharmaceuticals Corporation) and tacrolimus (TAC, FK506, Prograf; Astellas Pharmaceuticals Inc). Until recently, CSA was the mainstay of maintenance therapy, however, the 2009 ISHLT pediatric heart update reported that 58% of patients were started on TAC compared with 38% on CSA [32]. This shift in treatment regimen is likely multifactorial. While several studies have demonstrated equivalent graft survival between the two drugs [99, 100], CSA has a less favorable cosmetic side effect profile and is associated with more lipid derangements. In addition, a recent meta-analysis found decreased risk of acute rejection with TAC compared to CSA [101], although other trials have failed to confirm this finding [100, 102]. Even though there may be no difference in *de-novo* rejection rates when comparing the 2 agents, conversion from CSA to TAC may be useful in controlling recurrent or treatment-refractory rejection [99, 100, 103].

### Cell Cycle Inhibitors/Antiproliferatives

Azathioprine’s (AZA) active metabolite is converted into a purine analog (Table **4**). When incorporated into nuclear DNA, this metabolite inhibits DNA synthesis and subsequent T- and B-cell proliferation [104]. AZA is typically used in conjunction with a CNI in maintenance therapy and is effective in preventing rejection when used in combination with CSA and steroids [38]. AZA has largely been replaced by newer agents, although it is still used by some clinicians when patients develop intolerable side effects from newer antiproliferative agents [94].

Mycophenolate mofetil (MMF, Cellcept; Roche Laboratories, New Jersey, USA) has replaced AZA in current maintenance regimens (Table **4**). Nearly 60% of patients started on cell cycle inhibitors are given MMF compared to AZA in 21% [32]. Mycophenolate is a noncompetitive inhibitor of inosine monophosphate dehydrogenase, a key enzyme in *de novo* guanine nucleotide production. In proliferating lymphocytes, the only pathway for purine synthesis is *de novo* production, while in other cell lines, either salvage pathways or *de novo* synthesis can be used. Thus, mycophenolate blocks lymphocyte proliferation without inhibiting other cell lines [104]. MMF is a pro-drug that is quickly metabolized to mycophenolic acid (MPA) in the patient. When compared to AZA, MMF has been shown to improve survival, reduce acute rejection rates, and decrease the incidence of coronary vasculopathy in adults [105-107]. In children, MMF allows CNI dosing to be decreased which improves renal function without increasing rejection risk [108]. Because MPA levels have been correlated with rejection risk, MMF dosing should be concentration-driven rather than dose-driven since there is not a linear relationship between MPA trough level and MMF dose in children and young adults. First year MPA troughs <2.5 µg/mL have been associated with increased rejection [109]. Recently introduced is an enteric-coated formulation of mycophenolic acid (Myfortic; Novartis Pharmaceuticals Corporation, New Jersey, USA). This formulation appears equally efficacious with less gastrointestinal toxicity compared to MMF in adults, although no similar comparative pediatric data is yet available [110]. 

### mTOR Inhibitors

Increasingly, sirolimus (RAPA, Rapamycin, Rapamune, Wyeth) is being used in pediatric transplantation (Table **4**). RAPA inhibits the enzyme kinase *mammalian target of rapamycin* (mTOR). mTOR phosphorylates cell-cycle regulatory proteins involved in the T-cell proliferation, disrupts growth and differentiation of B- and T-cell lymphocytes [104] and inhibits vascular smooth muscle proliferation [94]. Sirolimus is nephrotoxic, although much less so than CNIs [94]. In adults, sirolimus has been shown to decrease CNI-induced renal insufficiency [111] and attenuate the development of coronary vasculopathy [112, 113]. However, a recent adult cohort study of CNI-free, primary mTOR inhibitor immune suppression in the immediate post-operative period demonstrated CNI-free regimens had increased early acute rejection, increased bacterial and fungal infections, and increased pleural effusions when compared to CNI or CNI+mTOR regimens [114]. In the pediatric literature, small studies have shown sirolimus to be effective in allowing lower doses of CNI which reduces CNI side effects, including renal toxicity, and as an adjunct treatment for rejection [115, 116]. Pediatric safety and efficacy for sirolimus remains poorly established, although a small retrospective study demonstrated no increase in hyperlipidemia, rejection, or mortality after converting to a lower CNI dose regimen with adjunctive sirolimus [117]. Everolimus (Certican, Novartis) is an alternative mTOR inhibitor which has a similar side effect profile to sirolimus and has gained wide acceptance with adult transplant physicians, but thus far has limited application in pediatrics.

### Corticosteroids

Corticosteroids (steroids) have roles in induction, maintenance therapy, and the treatment of rejection [38]. Steroids inhibit the transcription factors activator protein-1 and nuclear factor kappa-B, which both have important roles in the production of cytokines including IL-1 and IL-2, GM-CSF, TNF-alpha, growth factors, gamma interferon, CD40 ligand, and others [94, 104]. These actions limit the number, function, and distribution of white blood cells and provide broad, non-specific, immune suppression. Steroids are typically initiated during and immediately after surgery in high doses and subsequently tapered to lower maintenance dosing. Because of their side effect profile and long-term morbidities, many clinicians attempt to wean steroids off within several months of transplant depending on rejection history. Nonetheless, according to the most recent international society report, nearly 40% of pediatric patients still receive prednisone 5 years post-transplant [32]. 

With the introduction of new immunsuppressive agents, “steroid avoidance”, or early, rapid steroid weaning is increasingly targeted. A typical approach consists of 3-7 days of intravenous steroids in the peri-operative period followed by a rapid and complete taper off. Often these patients receive induction with Thymoglobulin followed by maintenance immunosuppression with tacrolimus or cyclosporine in conjunction with an anti-proliferative agent, commonly mycophenolate mofetil. Using this approach in appropriately selected patients, multiple studies have demonstrated that steroid-free therapy provides survival and rejection rates similar to patients who continue to receive maintenance corticosteroid therapy [82, 118, 119]. Unfortunately, not all patients tolerate steroid avoidance and some require steroid re-initiation secondary to late (>1 month post-transplant) or recurrent rejection [82]. For patients in whom avoidance was not tolerated or attempted, steroid withdrawal has been consistently and successfully implemented over the last decade as a strategy to decrease the adverse late-effects of steroids. Patients who are maintained on longer-term (> 5 years) steroids may be unable to taper off at all and seem to be at increased risk for late rejection when tapered off particularly in older recipients who may be more steroid dependent [120]. 

## OUTCOME

Overall peri-operative and short-term survival after pediatric transplant is good; >80% of patients survive to 1 year [32]. However, multiple studies have identified risk factors for mortality following pediatric heart transplant. Tjang and colleagues reported a single center series of 116 patients post-transplant and found that recipient age <1 year, recipient body height and body surface area, pre-transplant congenital heart disease, male donor, donor body height and surface area, and cardiopulmonary bypass time >210 minutes were associated with increased 30-day mortality. Multivariate analysis determined that male donor and prolonged bypass time were independent risk factors for 30-day mortality [121]. 

A recent study by Davies re-examined high-risk criteria for pediatric transplant which have traditionally included pulmonary vascular resistance (PVR) >6 Uxm^2^, renal failure, presence of hepatitis C antibody, donor/recipient weight ratio >0.7, PRA >40%, retransplantation, and age <1 year. To better assess if and to what extend these risk factors influenced early survival, these investigators reviewed 3502 transplant patients aged <21 years identified through the United Network for Organ Sharing (UNOS) database. They determined that mortality at 30 days and 1 year was indeed higher in patients with high-risk criteria. However, perhaps related to improvements in peri-operative management, increased PVR, retransplantation, and high PRA did not independently predict increased mortality. Of the traditional high-risk criteria, only renal failure was a risk factor for early mortality, but, factors including pre-operative ECMO, earlier era of transplant and pre-transplant congenital heart disease each added incremental risk [122]. 

Multiple studies have cited congenital heart disease (CHD) as an early mortality risk [19, 33, 121, 123]. However, the spectrum of CHD is broad. To better understand the factors that contribute to poor outcome in CHD patients, Lamour *et al* studied 488 patients transplanted with variable types of CHD including single ventricle lesions, d- and l-transposition of the great arteries, right ventricular outflow tract obstruction, atrial and ventricular septation defects and others; 93% of the patients had at least 1 cardiac surgery prior to transplant. Overall, patients with CHD had significantly lower survival at 3 months post-transplant than did cardiomyopathy patients. Risk factors for death were older recipient and donor ages and prolonged ischemic time. Fontan palliated-patients had significantly increased risk of death compared to other CHD after transplant [123]. Although the reasons for this decreased survival are multifactorial, multiple previous intra-thoracic surgeries and elevated pulmonary vascular resistance due to nature of Fontan-physiology are likely two of the main causes [123, 124]. Interestingly, the conditional risk of death for CHD patients who were alive at 3 months post-transplant was not different from pediatric patients with cardiomypathy suggesting that additional unidentified perioperative factors in the CHD group contribute to death [123]. Several smaller studies corroborate similar survival between CHD and non-CHD pediatric patients [125, 126]. In addition to an increased risk of allosensitization and elevated PVR that may have been underestimated by pre-operative assessment, complex anatomy often requires challenging vascular reconstructions and complicates surgical graft anastamoses. Furthermore, since many of these patients have had previous sternotomies, re-operation is technically more difficult and the risks of bleeding are significantly increased. A thorough understanding of the peri-operative factors that may affect short-term outcome in this group is therefore essential to ensure the best short and intermediate term survival.

## Figures and Tables

**Table 1:** ISHLT Guidelines for Post-Heart Transplant Monitoring [1]

Post-operative 12-lead ECG	Invasive arterial pressure monitoring
Right atrial or central venous pressure monitoring	Left atrial of pulmonary artery wedge pressure monitoring
Intermittent measures of cardiac output	Arterial oxygen saturation monitoring
Intra-operative transesophageal echocardiogram	Continuous assessment of urinary output

**Table 2:** Transfusion Guidelines for the ABO-Incompatible Patient [34]

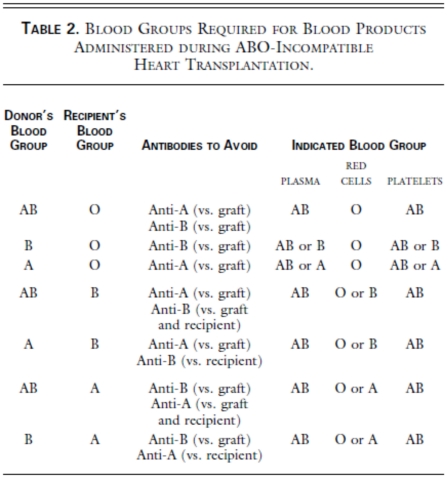

**Table 3:** Summary of Induction Agents [93, 94, 127]

Class / Drug	Dosing	Side Effects
***Polyclonal Anti-thymocyte Globulin***		
*Equine ATG*	After subQ test dose, 7-15 mg/kg/d by slow IV infusion for 5-7 days	Rash, fever, hypotension, serum sickness (with equine
*Rabbit ATG*	1.5 mg/kg/d for 3–7 days post-transplant by slow IV infusion	preparation), anaphylaxis, cytokine release syndrome
***IL-2 Receptor Antibodies***		
*Basiliximab*	12 mg/m^2^ up to 20 mg per dose in infused over 30 min on day 0 and day 4 post transplantation	Risk of hypersensitivity, anaphylaxis
*Daclizumab*	1 mg/kg IV perioperatively and then every 2 weeks, for a total of five doses	Risk of hypersensitivity, anaphylaxis

**Table 4:** Common Maintenance Immunosuppressive Agents [94, 104, 127, 128]

Class / Drug	Dosing	Side Effects
***Calcineurin Inhibitors***		
*Cyclosporine*	Based on goal trough levels; usual requirement 4-15 mg/kg/day divided twice daily	Nephrotoxicity, hypertension, hirsutism, gingival hypertrophy, hyperlipidemia, hyperkalemia, hypomagnesemia, seizures, encephalopathy
*Tacrolimus*	Based on goal trough levels; usual requirement 0.05- 0.3 mg/kg/day divided twice daily	Nephrotoxicity, hypertension, diabetes mellitus, alopecia, hyperkalemia, hypomagnesemia, headaches, paresthesias, seizures, encephalopathy
***Anti-proliferatives***		
*Azathioprine*	Based on white blood cell counts; usual requirement 1 to 3 mg/kg/day	Leukopenia, anemia, megaloblastic thrombocytopenia, pancreatitis, hepatitis, nausea, vomiting, diarrhea, anorexia, neoplasia
*Mycophenolate Mofetil*	25–50 mg/kg/day or 1,200 mg/m^2^/day divided twice daily; may target MPA trough levels 1.5-2	Nausea, vomiting, diarrhea, abdominal pain, anorexia, anemia, leukopenia
***m-TOR Inhibitors***		
*Sirolimus*	Based on goal trough levels; usual requirement 1–3 mg/m^2^/day	Hyperlipidemia, mucosal ulceration, anemia, thrombocytopenia, leukopenia, arthralgias, impaired wound healing, nephrotoxicity
*Everolimus*	Based on goal trough levels; usual requirement is 0.8mg/m^2^/day	Hyperlipidemia, hypertension stomatitis, anemia, thrombocytopenia, leukopenia, fatigue, impaired wound healing, nephrotoxicity
***Corticosteroids***		
*Prednisone*	Significant institutional variation; typical maintenance dose 0.05- 0.3 mg/kg/day	Hypertension, hyperlipidemia, diabetes, growth retardation, osteoporosis, increased infections, weight gain, adrenal suppression, cataracts, glaucoma, acne, headaches, pseudotumor cerebri
